# Dr. Troillet on the Cicatrization of Intestinal Ulcers

**Published:** 1826-07

**Authors:** 


### CICATRIZATION OF INTESTINAL ULCERS.*

It is only of late years that medical men have learnt the extent of Nature's powers in healing ulcerations of the internal surface of the intestinal tube. Our readers are aware that Dr. Latham, in his interesting Account of the Disease at Millbank, gave some examples of this important process, and in the writings of Petit, (of the Hotel Dieu) Andral, Jun. and M. Billard, the subject has been more fully discussed. We have not seen any cases where the mechanism (if we may use the expression) of the formation and healing of intestinal ulcers is so well displayed as in the Paper of M. Troillet. We shall here, therefore, introduce a succinct account of two cases elucidating this subject.

*Case.* Rose Guillon, ætat. 21, was carried to the Hotel Dieu of Lyons, on the 7th October, 1823, being the 9th day of her illness, presenting the following symptoms—flushed face—conjunctivæ injected—eyes watery—strength prostrated—tongue red at the sides, and greyish-brown in the centre—epigastrium painful—abdomen somewhat distended, and very tender on pressure—diarrhœa—pulse quick and small—skin dry and hot. *Eight leeches to the epigastrium, followed by blisters to the legs—mucilaginous drink—fomentations to the abdomen.* The disease advanced to the highest degree of intensity during the second and third weeks, but towards the close of the fourth, there was a sensible amelioration. The lips lost their fuligenous appearance, the delirium ceased, the countenance improved, the motions became more

---

* Dr. Troillet. Journ. Gen. de Médécine.

natural, the fever diminished, and was scarcely perceptible at the beginning of November. Some syrup of cinchona was now exhibited, and the patient having expressed a desire for food, some bouillie was ordered, but the nurse exceeded her instructions, and over-fed the patient between the 5th and 12th of November. Vomiting now came on—the epigastrium became painful—delirium was renewed—the lips became black—and the patient sunk on the 19th of November.

*Dissection.* Tunica arachnoides unaltered—slight injection of the vessels of the pia mater—brain and spinal marrow of a firm consistence. Nothing particular in the chest. *Abdomen.* Stomach and intestines distended with gas. The internal surface of the former was pale, with reddish streaks near the pylorus. There were some red points in the jejunum, which became more numerous in the ileum, particularly near its termination, where there were evident traces of ulceration for the space of 18 inches. Nothing remarkable in the large intestines.

The ulcerations were of an oval or round form, varying in diameter from six to ten lines, with fringed edges, and surrounded with a brownish circle, beyond which the mucous membrane was sound. Their surfaces presented the following marks of incipient, advanced, and complete cicatrization. *Those* in the *first* state were covered with a fine pellicle, transparent even after being washed or scraped with the scalpel. It was in some degree moveable on the subjacent cellular substance. In other ulcerations, where the work of regeneration was more advanced, the pellicle was thicker, slightly opake in some parts of its surface, and amalgamated as it were, with the fringed edges of the sore. In those ulcers where cicatrization was nearly completed, the pellicle had acquired the thickness, the consistence, and the aspect of the common mucous membrane. In those parts where the healing process had advanced to the greatest degree, the fringed condition of the edges had entirely disappeared, the surrounding circle was of a faint colour, or in some places annihilated—and the mucous membrane was completely regenerated. These regenerations were more numerous at the upper part of the ulcerated portion of ileum. As the valve of the colon was approached, the whole surface of the intestine became altered in structure, crowded with ulcerations, and presenting but few marks of cicatrization.

*Case* 2. Ph. Delorme, aged 23 years, caught cold while travelling from Paris in a diligence. He became unwell, and about a fortnight afterwards (7th December, 1823) was received into the Hôtel Dieu, of Lyons, with the following symptoms: flushed face—general debility—cephalalgia—tinnitus aurium—insomnium—tongue white in the middle and red at the sides—epigastrium painful—diarrhœa—pulse quick—hot skin. *Diluents—low diet.* 8th, Belly painful and distended—diarrhœa troublesome—pulse hard and resisting. *Venesection to* 15 *ounces.* 9th, The same symptoms. 10th, Violent colicky pains and urgent diarrhœa. Eight leeches to the abdomen. 11th, Pain and distention of the abdomen abated. 12th. The same. 13th, The abdominal pains persist. *Ten leeches to the abdomen, followed by fomen-*

*tations and poultices. Mucilaginous drink.* 14th, The symptoms were ameliorated, but the fever had a marked diurnal paroxysm from this till the 18th. *Two grains of the sulphate of quinine were ordered per diem.* 20th, The patient is better—the diarrhœa has ceased—and strength is returning. 29th, The patient can walk about the hospital, and took an opportunity to indulge his appetite freely. The consequence was a violent accession of fever, and the abdominal pains returned. The fever and other bad symptoms continued during the following days, with swelling of the abdomen and dysenteric purging. 2d. January, delirium came on. He lingered till the 7th, when he died.

*Dissection.* No appearance of disease could be seen in the head or in the chest. The mucous membrane of the stomach was pale. The small intestines presented few traces of ulceration till they reached the lower part of the ileum, and there the morbid alterations existed in various degrees, which it is important to detail, as exemplifying the progress of these diseased conditions.

1$^{mo.}$ In the points which were merely inflamed, the mucous membrane was tumefied, red, softened, and presenting certain fungous spots of three or four lines in breadth. 2$^{ndo.}$ In the centres of some of these fungous spots there appeared dark specks as if caused by the effusion of a minute drop of blood from a vessel. 3$^{tio.}$ In the centres of others were seen a minute speck of ulceration, surrounded by a small black circle, and penetrating through the mucous coat. 4$^{to.}$ In other places, and especially in the lower portion of ileum, the ulcers were larger, extending to 8 or 10 lines in diameter. They were surrounded with a dark areola, and this areola was again encircled by a red halo. These ulcers, whose edges were perpendicular, were of a pale colour, and did not appear to go below the mucous membrane. The greater number of them were covered with a fine pellicle, in some transparent, in others opake and thickened, in others still, appearing quite regenerated into mucous membrane again, but yet encircled with a diminished halo of a dark colour.

*Remarks.* In both of these cases Nature seems to have unveiled her mode of proceeding in the *regenerative* process—in the second case the process of the *formation* of the ulcers is developed. But what is the cause of these ulcerations in the intestines? Inflammatory irritation, most people will answer. Yet we see great inflammation accompanied by great irritation, without any such effect being the result. Again we see aphthous ulcers in the mouth and throat, where the membrane is pale, and where there is no appearance of inflammatory action. M. Troillet observes, however, that the effects of irritation differ greatly in the different structures of the body. In the nutritive absorbents it produces disorganization—ulceration. In the exhalent absorbents, on the other hand, it produces morbid growth and tumours. These considerations, he thinks, explain the formation of ulcers in the intestines, without the necessity of actual inflammation in the parts. He doubts not, however, that inflammation sometimes accompanies the ulcerative process, as well as the growth of tumours. This takes place when the irritation is not bounded to the nutritive absorbents, but is extended to the nervous and capillary structures. In many instances, however, there is no evidence of inflammation, as in the cases under review, and in numerous morbid growths in other parts of the body.

We do not deem it necessary to detail M. Troillet's theory respecting the healing of intestinal ulcers. It is sufficient for our present purpose, that the fact of their healing is completely established by these and other instances. The fact, we conceive, is a very interesting one, and we are obliged to M. Troillet for the minute detail of the cases in this paper.

---

